# Supplementary material for: Integrated Pan-Cancer Analysis and Experimental Verification of the Roles of Retinoid-Binding Proteins in Breast Cancer
Source: Cancers (Basel). 2025 Nov 19;17(22):3706. doi: 10.3390/cancers17223706 (PMC12651014; doi:10.3390/cancers17223706)
Supplement: Supplementary file 1 [file cancers-17-03706-s001.zip › cancers-3948508-supplementary.pdf]

Supplementary Information

Pan-cancer Analysis of Retinoid-Binding Proteins: Implications for Prognosis and Tumor Microenvironment

Yuchu Xiang[1,2,†], Dan Du [1,2,†], Yaoxi Su[1,2], Linghong Guo[1,2,*], Siliang Chen[1,2,*]

1 Department of Dermatology & Venereology, West China Hospital, Sichuan University, Chengdu 610041, China; xiangyuchu@stu.scu.edu.cn (Y.X.); dudan.meishan@foxmail.com (D.D.); suyaoxi@wchscu.edu.cn (Y.S.)

2 Laboratory of Dermatology, Clinical Institute of Inflammation and Immunology, Frontiers Science Center for Disease-related Molecular Network, West China Hospital, Sichuan University, Chengdu 610041, China

† These authors contributed equally and share the first authorship

* Correspondence: linhom.guo@foxmail.com (L.G.); siliangchen220701@wchscu.edu.cn (S.C.)

Table S1. Top-10 marker genes.

	p_val	avg_log2FC	pct.1	pct.2	p_val_adj	cluster	gene
1	0	4.73894466	0.789	0.111	0	0	DST
2	0	3.90918309	0.884	0.222	0	0	KRT5
3	0	5.23559696	0.602	0.045	0	0	ACTG2
4	0	4.56285385	0.541	0.043	0	0	LAMA3
5	0	5.10431287	0.432	0.021	0	0	TNS4
6	0	5.92521548	0.415	0.009	0	0	COL17A1
7	0	4.23355312	0.366	0.023	0	0	DKK3
8	0	7.07830942	0.333	0.005	0	0	OXTR
9	0	4.27082667	0.305	0.018	0	0	CNN1
10	0	3.7413332	0.285	0.029	0	0	DLL1
11	0	6.82171179	0.792	0.03	0	1	DCN
12	0	7.09158299	0.644	0.098	0	1	MMP3
13	0	6.54538337	0.434	0.01	0	1	THBS2
14	0	8.68329354	0.397	0.004	0	1	DLK1
15	0	6.95280845	0.413	0.023	0	1	MMP10
16	0	6.43984408	0.386	0.008	0	1	PDGFRA
17	0	6.77879956	0.346	0.007	0	1	TAC1
18	0	8.36582764	0.305	0.002	0	1	DPT
19	0	7.82323214	0.259	0.003	0	1	RSPO3
20	0	8.25930633	0.254	0.001	0	1	WNT2
21	0	4.1443606	0.875	0.09	0	2	S100P
22	0	4.23790241	0.439	0.031	0	2	FGFBP2
23	0	4.34951401	0.42	0.035	0	2	LY6D
24	0	3.97591288	0.387	0.027	0	2	LEMD1
25	0	4.56093566	0.348	0.02	0	2	RHCG
26	0	5.66972269	0.316	0.016	0	2	SPRR2A
27	0	4.93408779	0.313	0.027	0	2	SBSN
28	0	4.54546782	0.282	0.015	0	2	PSORS1C2

29	0	3.96388751	0.259	0.017	0	2	VGLL1
30	0	6.11955722	0.258	0.019	0	2	KRTDAP
31	0	7.28662155	0.684	0.008	0	3	CD3D
32	0	7.17003496	0.63	0.007	0	3	CD2
33	0	7.26469136	0.544	0.012	0	3	CD69
34	0	7.13976987	0.523	0.006	0	3	TRBC2
35	0	7.17373909	0.489	0.006	0	3	TRBC1
36	0	7.33433344	0.468	0.008	0	3	CD7
37	0	7.65722641	0.312	0.002	0	3	CD3E
38	0	7.09288417	0.309	0.004	0	3	CTSW
39	0	7.24183962	0.277	0.004	0	3	LINC01871
40	0	7.18935689	0.27	0.003	0	3	TIGIT
41	0	3.80878539	0.557	0.07	0	4	ALDH1A3
42	0	3.52497426	0.673	0.223	0	4	MMP7
43	0	5.84333852	0.464	0.015	0	4	PIGR
44	0	4.83042116	0.424	0.03	0	4	SCGB3A1
45	0	5.20724004	0.384	0.014	0	4	CYP24A1
46	0	3.66989716	0.39	0.027	0	4	S100A1
47	0	3.14355002	0.391	0.064	0	4	KRT23
48	0	4.90123894	0.294	0.011	0	4	CCL28
49	0	4.55765558	0.298	0.019	0	4	LTF
50	0	4.49620167	0.278	0.012	0	4	SNORC
51	0	8.33098115	0.827	0.012	0	5	PCAT19
52	0	8.57636339	0.613	0.003	0	5	ADGRL4
53	0	7.7376142	0.416	0.004	0	5	PLVAP
54	0	8.20869897	0.401	0.005	0	5	CLDN5
55	0	9.10348875	0.395	0.006	0	5	SELE
56	0	8.1202972	0.349	0.002	0	5	ECSCR
57	0	8.88584476	0.316	0.001	0	5	CDH5
58	0	8.76457236	0.307	0.001	0	5	MYCT1
59	0	7.98234389	0.289	0.004	0	5	ACKR1
60	0	7.98290476	0.285	0.003	0	5	SOX17
61	0	5.30468936	0.692	0.026	0	6	BIRC5
62	0	5.31737459	0.601	0.022	0	6	CDK1
63	0	5.22947972	0.561	0.022	0	6	CENPF
64	0	5.43551419	0.534	0.017	0	6	CCNB2
65	0	5.28782743	0.493	0.015	0	6	TOP2A
66	0	5.33949171	0.484	0.014	0	6	MKI67
67	0	5.49698801	0.465	0.013	0	6	SPC25
68	0	5.454117	0.435	0.012	0	6	CDCA3
69	0	5.48606909	0.328	0.008	0	6	NUF2
70	0	5.32130379	0.307	0.009	0	6	GTSE1
71	0	5.03651941	0.833	0.177	0	7	MT1A
72	0	4.02727632	0.754	0.147	0	7	C11orf96
73	0	3.65585542	0.707	0.139	0	7	ADAMTS4
74	0	4.5095569	0.672	0.163	0	7	IL6
75	0	6.00283436	0.47	0.016	0	7	RGS5
76	0	4.10402947	0.509	0.078	0	7	PDK4
77	0	4.96769234	0.401	0.024	0	7	KCNE4
78	0	4.37000144	0.418	0.041	0	7	SSTR2
79	0	4.56641154	0.351	0.035	0	7	PROCR
80	0	5.2765551	0.308	0.014	0	7	PRKG1
81	0	9.25741775	0.811	0.014	0	8	LYZ

82	0	8.22667199	0.76	0.007	0	8	AIF1
83	0	9.84989801	0.537	0.003	0	8	C1QC
84	0	9.61950442	0.535	0.005	0	8	C1QA
85	0	9.42034289	0.512	0.005	0	8	C1QB
86	0	8.43408439	0.395	0.002	0	8	MS4A7
87	0	9.59077712	0.352	0.001	0	8	FPR3
88	0	8.38158652	0.332	0.001	0	8	MNDA
89	0	9.67125734	0.282	0.001	0	8	OLR1
90	0	8.7314966	0.268	0.001	0	8	MS4A4A
91	0	7.01931082	0.947	0.078	0	9	AZGP1
92	0	7.26516767	0.612	0.02	0	9	TFF3
93	0	8.44192806	0.59	0.003	0	9	TMC5
94	0	8.87320227	0.557	0.007	0	9	TFF1
95	0	5.35579124	0.527	0.021	0	9	TGM2
96	0	8.25604106	0.467	0.002	0	9	ANKRD30A
97	0	5.25809224	0.482	0.02	0	9	DNAJC12
98	0	7.48243878	0.451	0.004	0	9	TMEM45B
99	0	5.47305611	0.379	0.015	0	9	AGR2
100	1.2e-228	6.75592145	0.262	0.032	2.9e-224	9	MUCL1
101	0	10.716578	0.881	0.007	0	10	JCHAIN
102	0	11.5265269	0.889	0.024	0	10	IGHG3
103	0	11.5529146	0.839	0.015	0	10	IGHG1
104	0	11.3684358	0.864	0.096	0	10	IGKC
105	0	12.3278315	0.72	0.002	0	10	IGHG2
106	0	11.0931597	0.706	0.004	0	10	IGHG4
107	0	12.3035008	0.701	0.001	0	10	IGHGP
108	0	12.0225612	0.734	0.056	0	10	IGLC2
109	0	13.0427613	0.557	0.017	0	10	IGLC3
110	0	13.13045	0.28	0	0	10	IGLC7

Table S2. Percentage for RBP4 positive cells.

cell_type	sample	condition	n_cells	n_RBP4_pos	prop_RBP4_pos
B Cells	Normal1	Normal	1	0	0
B Cells	Normal2	Normal	3	0	0
B Cells	Normal3	Normal	4	0	0
B Cells	Normal4	Normal	1	0	0
B Cells	Normal5	Normal	1	0	0
B Cells	TNBC1	TNBC	202	0	0
B Cells	TNBC2	TNBC	48	2	0.041667
B Cells	TNBC3	TNBC	88	0	0
B Cells	TNBC4	TNBC	13	0	0
Basal Cells	Normal1	Normal	973	4	0.004111
Basal Cells	Normal2	Normal	1887	17	0.009009
Basal Cells	Normal3	Normal	101	0	0
Basal Cells	Normal4	Normal	368	2	0.005435
Basal Cells	Normal5	Normal	1771	9	0.005082
Basal Cells	TNBC1	TNBC	5	0	0
Basal Cells	TNBC3	TNBC	5	0	0
Basal Cells	TNBC4	TNBC	14	0	0
Endothelial Cells	Normal1	Normal	177	10	0.056497
Endothelial Cells	Normal2	Normal	199	16	0.080402

Endothelial Cells	Normal3	Normal	383	20	0.052219
Endothelial Cells	Normal4	Normal	108	7	0.064815
Endothelial Cells	Normal5	Normal	1081	235	0.217391
Endothelial Cells	TNBC1	TNBC	12	0	0
Endothelial Cells	TNBC2	TNBC	11	0	0
Endothelial Cells	TNBC3	TNBC	5	0	0
Endothelial Cells	TNBC4	TNBC	4	0	0
Fibroblasts	Normal1	Normal	537	5	0.009311
Fibroblasts	Normal2	Normal	871	10	0.011481
Fibroblasts	Normal3	Normal	413	0	0
Fibroblasts	Normal4	Normal	1296	4	0.003086
Fibroblasts	Normal5	Normal	1137	3	0.002639
Fibroblasts	TNBC1	TNBC	132	0	0
Fibroblasts	TNBC2	TNBC	92	0	0
Fibroblasts	TNBC3	TNBC	10	0	0
Fibroblasts	TNBC4	TNBC	17	0	0
Luminal Epithelial Cells	Normal1	Normal	1003	11	0.010967
Luminal Epithelial Cells	Normal2	Normal	453	1	0.002208
Luminal Epithelial Cells	Normal3	Normal	412	1	0.002427
Luminal Epithelial Cells	Normal4	Normal	92	0	0
Luminal Epithelial Cells	Normal5	Normal	236	2	0.008475
Luminal Epithelial Cells	TNBC1	TNBC	20	0	0
Luminal Epithelial Cells	TNBC2	TNBC	163	1	0.006135
Luminal Epithelial Cells	TNBC3	TNBC	16	0	0
Luminal Epithelial Cells	TNBC4	TNBC	30	0	0
Luminal Secretory Cells	Normal1	Normal	354	1	0.002825
Luminal Secretory Cells	Normal2	Normal	133	0	0
Luminal Secretory Cells	Normal3	Normal	12	0	0
Luminal Secretory Cells	Normal4	Normal	40	0	0
Luminal Secretory Cells	Normal5	Normal	165	1	0.006061
Luminal Secretory Cells	TNBC1	TNBC	1	0	0
Luminal Secretory Cells	TNBC4	TNBC	29	0	0
Macrophages	Normal1	Normal	49	0	0
Macrophages	Normal2	Normal	57	0	0
Macrophages	Normal3	Normal	6	0	0
Macrophages	Normal4	Normal	15	0	0
Macrophages	Normal5	Normal	8	0	0
Macrophages	TNBC1	TNBC	373	1	0.002681
Macrophages	TNBC2	TNBC	529	2	0.003781
Macrophages	TNBC3	TNBC	150	0	0
Macrophages	TNBC4	TNBC	57	4	0.070175
Pericytes	Normal1	Normal	234	3	0.012821
Pericytes	Normal2	Normal	279	1	0.003584
Pericytes	Normal3	Normal	240	1	0.004167
Pericytes	Normal4	Normal	251	2	0.007968
Pericytes	Normal5	Normal	394	4	0.010152
Pericytes	TNBC1	TNBC	9	0	0
Pericytes	TNBC2	TNBC	27	0	0
Pericytes	TNBC3	TNBC	8	0	0
Pericytes	TNBC4	TNBC	21	0	0
Proliferating Tumor Cells	Normal1	Normal	4	0	0
Proliferating Tumor Cells	Normal2	Normal	6	0	0
Proliferating Tumor Cells	Normal3	Normal	36	0	0

Proliferating Tumor Cells	TNBC1	TNBC	147	0	0
Proliferating Tumor Cells	TNBC2	TNBC	1583	36	0.022742
Proliferating Tumor Cells	TNBC3	TNBC	12	0	0
Proliferating Tumor Cells	TNBC4	TNBC	18	0	0
T Cells	Normal1	Normal	75	0	0
T Cells	Normal2	Normal	94	0	0
T Cells	Normal3	Normal	2	0	0
T Cells	Normal4	Normal	33	0	0
T Cells	Normal5	Normal	8	0	0
T Cells	TNBC1	TNBC	1251	0	0
T Cells	TNBC2	TNBC	108	0	0
T Cells	TNBC3	TNBC	416	0	0
T Cells	TNBC4	TNBC	839	2	0.002384
Tumor Epithelial Cell	TNBC1	TNBC	204	3	0.014706
Tumor Epithelial Cell	TNBC2	TNBC	3982	61	0.015319
Tumor Epithelial Cell	TNBC3	TNBC	4	0	0

Table S3. Percentage for RBP7 positive cells.

cell_type	sample	condition	n_cells	n_RBP7_pos	prop_RBP7_pos
B Cells	Normal1	Normal	1	0	0
B Cells	Normal2	Normal	3	0	0
B Cells	Normal3	Normal	4	0	0
B Cells	Normal4	Normal	1	0	0
B Cells	Normal5	Normal	1	0	0
B Cells	TNBC1	TNBC	202	3	0.014851
B Cells	TNBC2	TNBC	48	7	0.145833
B Cells	TNBC3	TNBC	88	0	0
B Cells	TNBC4	TNBC	13	0	0
Basal Cells	Normal1	Normal	973	27	0.027749
Basal Cells	Normal2	Normal	1887	20	0.010599
Basal Cells	Normal3	Normal	101	7	0.069307
Basal Cells	Normal4	Normal	368	9	0.024457
Basal Cells	Normal5	Normal	1771	40	0.022586
Basal Cells	TNBC1	TNBC	5	1	0.2
Basal Cells	TNBC3	TNBC	5	0	0
Basal Cells	TNBC4	TNBC	14	1	0.071429
Endothelial Cells	Normal1	Normal	177	123	0.694915
Endothelial Cells	Normal2	Normal	199	86	0.432161
Endothelial Cells	Normal3	Normal	383	187	0.488251
Endothelial Cells	Normal4	Normal	108	56	0.518519
Endothelial Cells	Normal5	Normal	1081	474	0.438483
Endothelial Cells	TNBC1	TNBC	12	1	0.083333
Endothelial Cells	TNBC2	TNBC	11	3	0.272727
Endothelial Cells	TNBC3	TNBC	5	2	0.4
Endothelial Cells	TNBC4	TNBC	4	2	0.5
Fibroblasts	Normal1	Normal	537	17	0.031657
Fibroblasts	Normal2	Normal	871	9	0.010333
Fibroblasts	Normal3	Normal	413	8	0.01937
Fibroblasts	Normal4	Normal	1296	33	0.025463
Fibroblasts	Normal5	Normal	1137	9	0.007916
Fibroblasts	TNBC1	TNBC	132	1	0.007576

Fibroblasts	TNBC2	TNBC	92	6	0.065217
Fibroblasts	TNBC3	TNBC	10	0	0
Fibroblasts	TNBC4	TNBC	17	0	0
Luminal Epithelial Cells	Normal1	Normal	1003	316	0.315055
Luminal Epithelial Cells	Normal2	Normal	453	52	0.11479
Luminal Epithelial Cells	Normal3	Normal	412	46	0.11165
Luminal Epithelial Cells	Normal4	Normal	92	16	0.173913
Luminal Epithelial Cells	Normal5	Normal	236	44	0.186441
Luminal Epithelial Cells	TNBC1	TNBC	20	2	0.1
Luminal Epithelial Cells	TNBC2	TNBC	163	27	0.165644
Luminal Epithelial Cells	TNBC3	TNBC	16	0	0
Luminal Epithelial Cells	TNBC4	TNBC	30	0	0
Luminal Secretory Cells	Normal1	Normal	354	36	0.101695
Luminal Secretory Cells	Normal2	Normal	133	7	0.052632
Luminal Secretory Cells	Normal3	Normal	12	2	0.166667
Luminal Secretory Cells	Normal4	Normal	40	3	0.075
Luminal Secretory Cells	Normal5	Normal	165	30	0.181818
Luminal Secretory Cells	TNBC1	TNBC	1	0	0
Luminal Secretory Cells	TNBC4	TNBC	29	0	0
Macrophages	Normal1	Normal	49	0	0
Macrophages	Normal2	Normal	57	1	0.017544
Macrophages	Normal3	Normal	6	0	0
Macrophages	Normal4	Normal	15	0	0
Macrophages	Normal5	Normal	8	0	0
Macrophages	TNBC1	TNBC	373	11	0.029491
Macrophages	TNBC2	TNBC	529	39	0.073724
Macrophages	TNBC3	TNBC	150	7	0.046667
Macrophages	TNBC4	TNBC	57	1	0.017544
Pericytes	Normal1	Normal	234	5	0.021368
Pericytes	Normal2	Normal	279	9	0.032258
Pericytes	Normal3	Normal	240	7	0.029167
Pericytes	Normal4	Normal	251	7	0.027888
Pericytes	Normal5	Normal	394	15	0.038071
Pericytes	TNBC1	TNBC	9	0	0
Pericytes	TNBC2	TNBC	27	0	0
Pericytes	TNBC3	TNBC	8	0	0
Pericytes	TNBC4	TNBC	21	0	0
Proliferating Tumor Cells	Normal1	Normal	4	0	0
Proliferating Tumor Cells	Normal2	Normal	6	0	0
Proliferating Tumor Cells	Normal3	Normal	36	5	0.138889
Proliferating Tumor Cells	TNBC1	TNBC	147	17	0.115646
Proliferating Tumor Cells	TNBC2	TNBC	1583	926	0.584965
Proliferating Tumor Cells	TNBC3	TNBC	12	0	0
Proliferating Tumor Cells	TNBC4	TNBC	18	0	0
T Cells	Normal1	Normal	75	1	0.013333
T Cells	Normal2	Normal	94	2	0.021277
T Cells	Normal3	Normal	2	0	0
T Cells	Normal4	Normal	33	0	0
T Cells	Normal5	Normal	8	0	0
T Cells	TNBC1	TNBC	1251	4	0.003197
T Cells	TNBC2	TNBC	108	2	0.018519
T Cells	TNBC3	TNBC	416	0	0
T Cells	TNBC4	TNBC	839	0	0

Tumor Epithelial Cell	TNBC1	TNBC	204	113	0.553922
Tumor Epithelial Cell	TNBC2	TNBC	3982	1726	0.433451
Tumor Epithelial Cell	TNBC3	TNBC	4	0	0

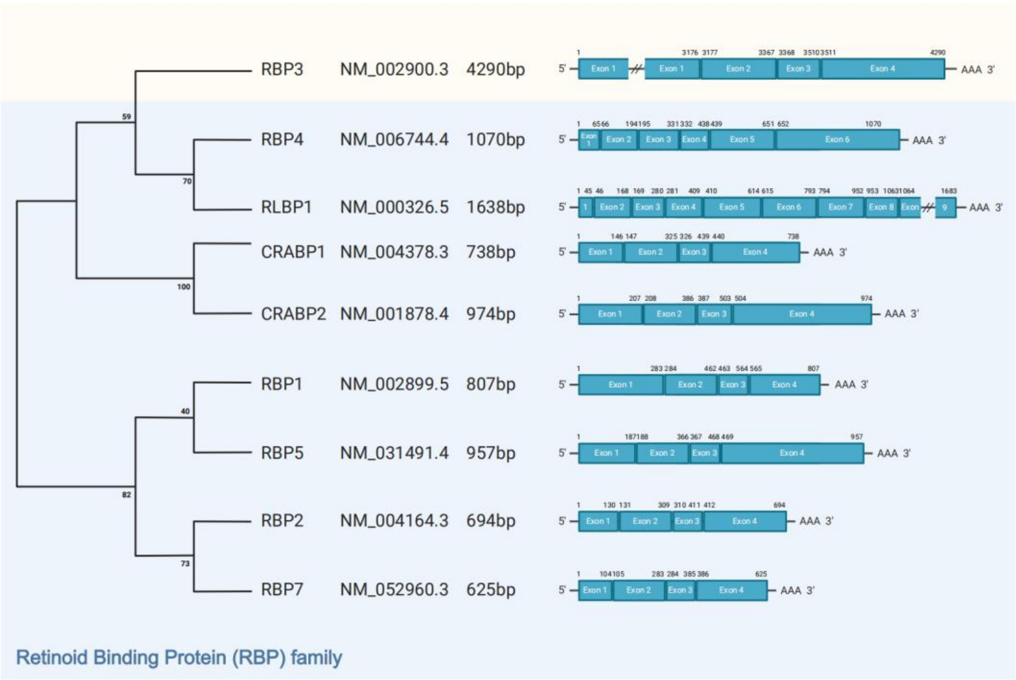

Figure S1. Phylogenetic relationships, conserved protein motifs and gene structures of the RBP family members.

A

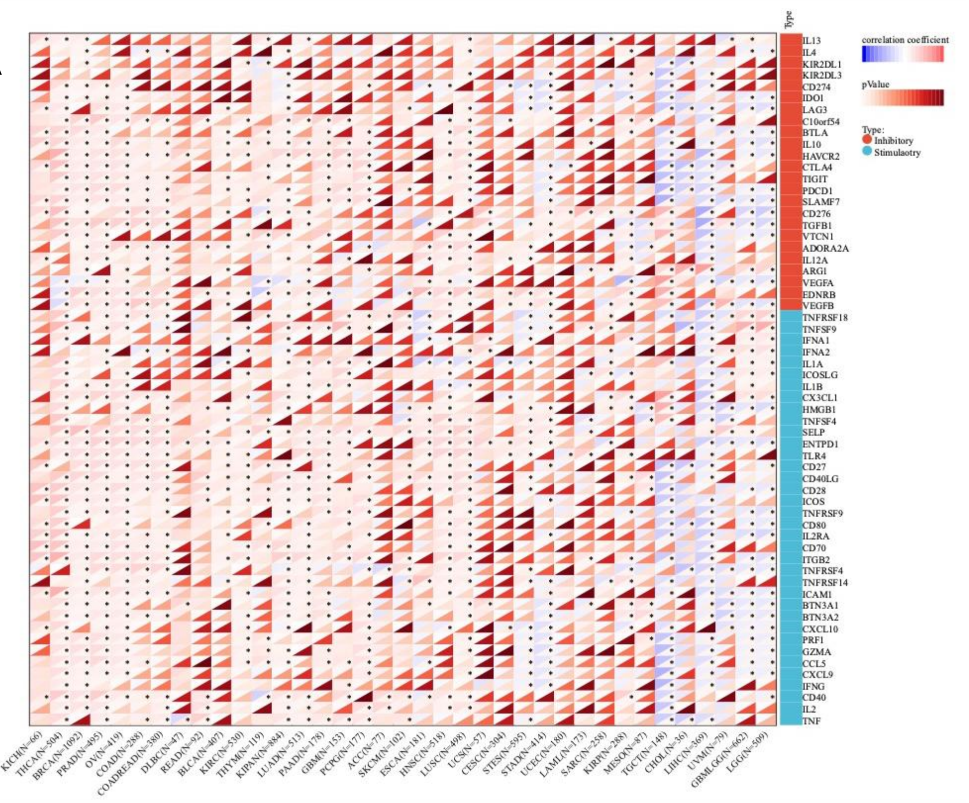

Type
correlation coefficient
pValue
Type:
Inhibitory
Stimulaotry
IL13
IL4
KIR2DL1
KIR2DL3
CD274
IDO1
LAG3
C10orf54
BTLA
IL10
HAVCR2
CTLA4
TIGIT
PDCD1
SLAMF7
CD276
TGFB1
VTCN1
ADORA2A
IL12A
ARG1
VEGFA
EDNRB
VEGFB
TNFRSF18
TNFSF9
IFNA1
IFNA2
IL1A
ICOSLG
IL1B
CX3CL1
HMGB1
TNFSF4
SELP
ENTPD1
TLR4
CD27
CD40LG
CD28
ICOS
TNFRSF9
CD80
IL2RA
CD70
ITGB2
TNFRSF4
TNFRSF14
ICAM1
BTN3A1
BTN3A2
CXCL10
PRF1
GZMA
CCL5
CXCL9
IFNG
CD40
IL2
TNF
KICH(N=66)
THCA(N=504)
BRCA(N=1092)
PRAD(N=495)
OV(N=419)
COAD(N=288)
COADREAD(N=380)
DLBC(N=47)
READ(N=92)
BLCA(N=407)
KIRC(N=530)
THYM(N=119)
KIPAN(N=884)
LUAD(N=513)
PAAD(N=178)
GBM(N=153)
PCPG(N=177)
ACC(N=77)
SKCM(N=102)
ESCA(N=181)
HNSC(N=518)
LUSC(N=498)
UCS(N=57)
CESC(N=304)
STES(N=595)
STAD(N=414)
UCEC(N=180)
LAML(N=173)
SARC(N=258)
KIRP(N=288)
MESO(N=87)
TGCT(N=148)
CHOL(N=36)
LIHC(N=369)
UVM(N=79)
GBMLGG(N=662)
LGG(N=509)

B

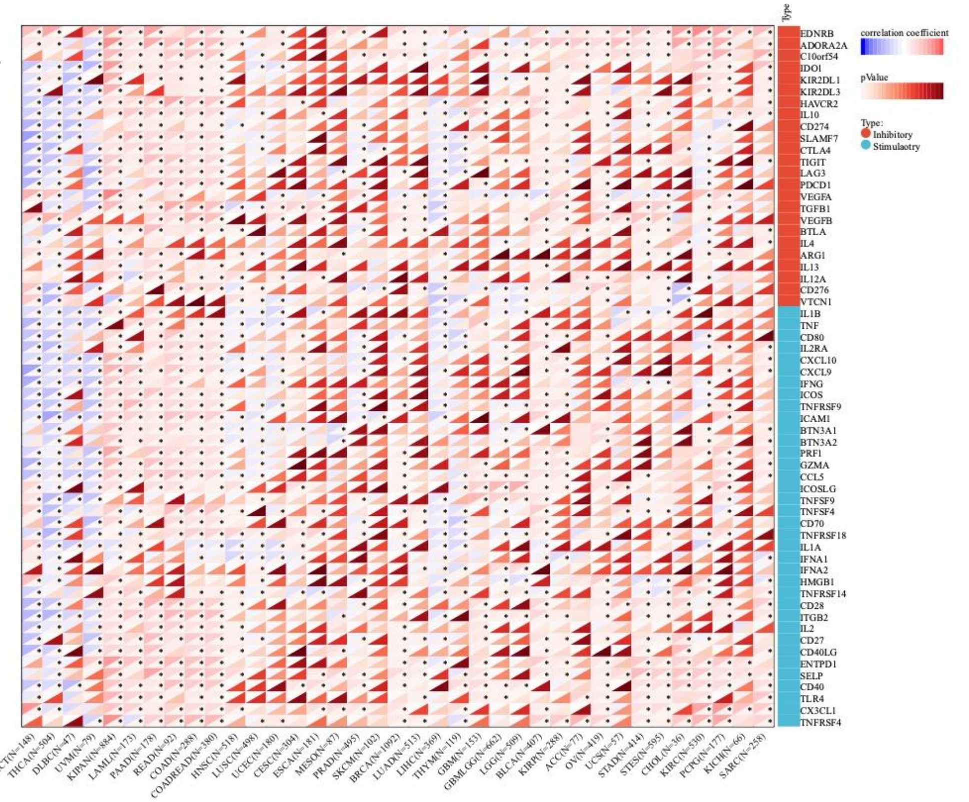

Type
correlation coefficient
pValue
Type:
Inhibitory
Stimulaotry
EDNRB
ADORA2A
C10orf54
IDO1
KIR2DL1
KIR2DL3
HAVCR2
IL10
CD274
SLAMF7
CTLA4
TIGIT
LAG3
PDCD1
VEGFA
TGFB1
VEGFB
BTLA
IL4
ARG1
IL13
IL12A
CD276
VTCN1
IL1B
TNF
CD80
IL2RA
CXCL10
CXCL9
IFNG
ICOS
TNFRSF9
ICAM1
BTN3A1
BTN3A2
PRF1
GZMA
CCL5
ICOSLG
TNFSF9
TNFSF4
CD70
TNFRSF18
IL1A
IFNA1
IFNA2
HMGB1
TNFRSF14
CD28
ITGB2
IL2
CD27
CD40LG
ENTPD1
SELP
CD40
TLR4
CX3CL1
TNFRSF4
TGCT(N=148)
THCA(N=504)
DLBC(N=47)
UVM(N=79)
KIPAN(N=884)
LAML(N=173)
PAAD(N=178)
READ(N=92)
COAD(N=288)
COADREAD(N=380)
HNSC(N=518)
LUSC(N=498)
UCEC(N=180)
CESC(N=304)
ESCA(N=181)
MESO(N=87)
PRAD(N=495)
SKCM(N=102)
BRCA(N=1092)
LUAD(N=513)
LIHC(N=369)
THYM(N=119)
GBM(N=153)
GBMLGG(N=662)
LGG(N=509)
BLCA(N=407)
KIRP(N=288)
ACC(N=77)
OV(N=419)
UCS(N=57)
STAD(N=414)
STES(N=595)
CHOL(N=36)
KIRC(N=530)
PCPG(N=177)
KICH(N=66)
SARC(N=258)

Figure S2. The relationship between RBP4/RBP7 and immune checkpoints. (A) Heatmap illustrating the relationship between RBP4 and known immune checkpoints. (B) Heatmap illustrating the relationship between RBP7 and known immune checkpoints. $*$P< 0.05, $**$P< 0.01, and $***$P< 0.001.

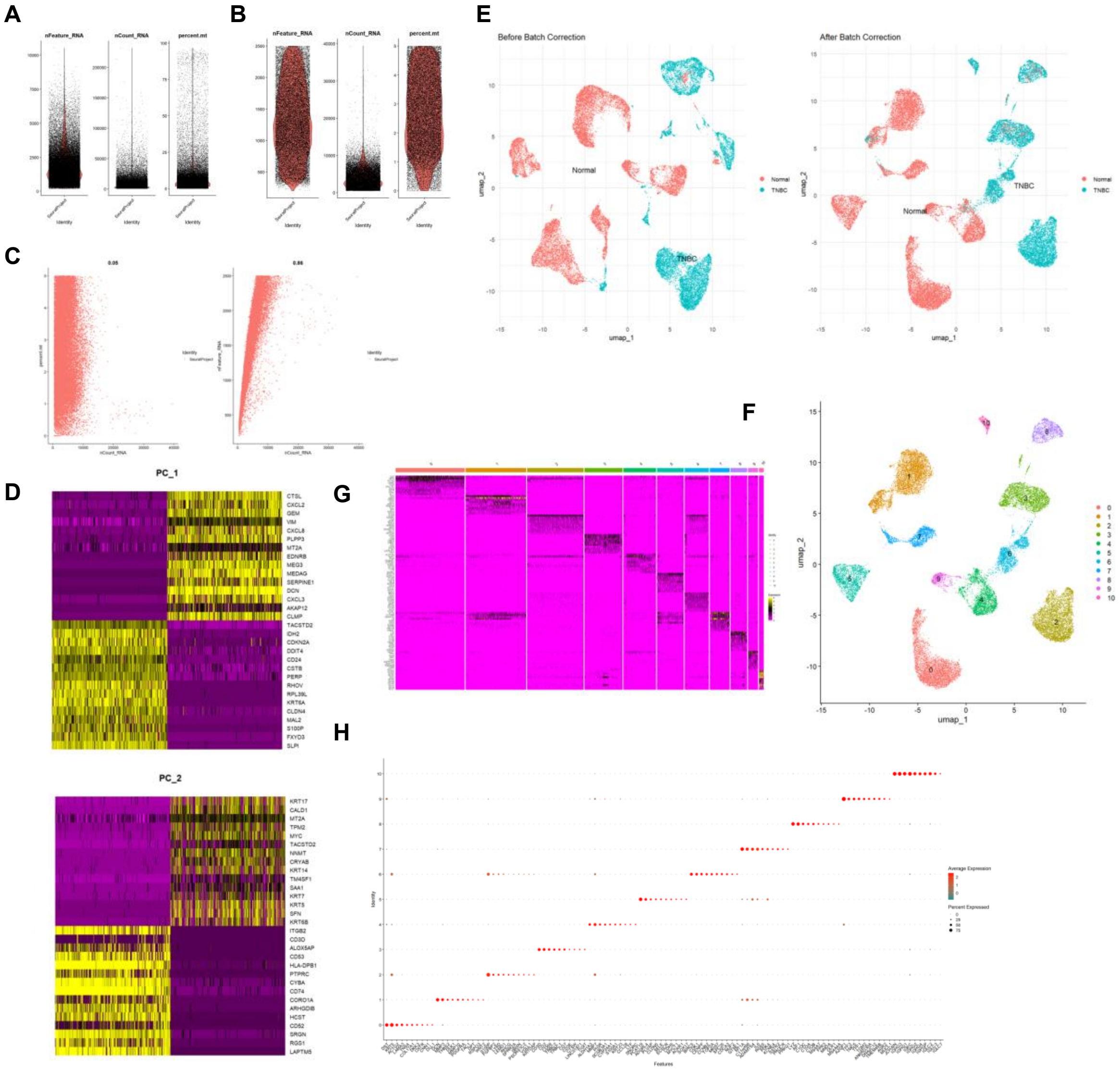

Figure S3. Expression Profiles of RBP4 and RBP7 in Single-Cell Transcriptome Analysis. (A-B) Quality control metrics before and after filtering, including nFeature_RNA, nCount_RNA, and the proportion of mitochondrial genes. (C) Scatter plot showing the positive correlation between nCount_RNA and nFeature_RNA after filtering. (D) Heatmap showing representative genes for PC1 and PC2 following dimensionality reduction. (E) UMAP before and after Batch correction. (F) UMAP plot

illustrating 10 distinct cell clusters after clustering analysis. (G-H) Heatmap and bubble ploto f the top 10 marker genes for each cluster.

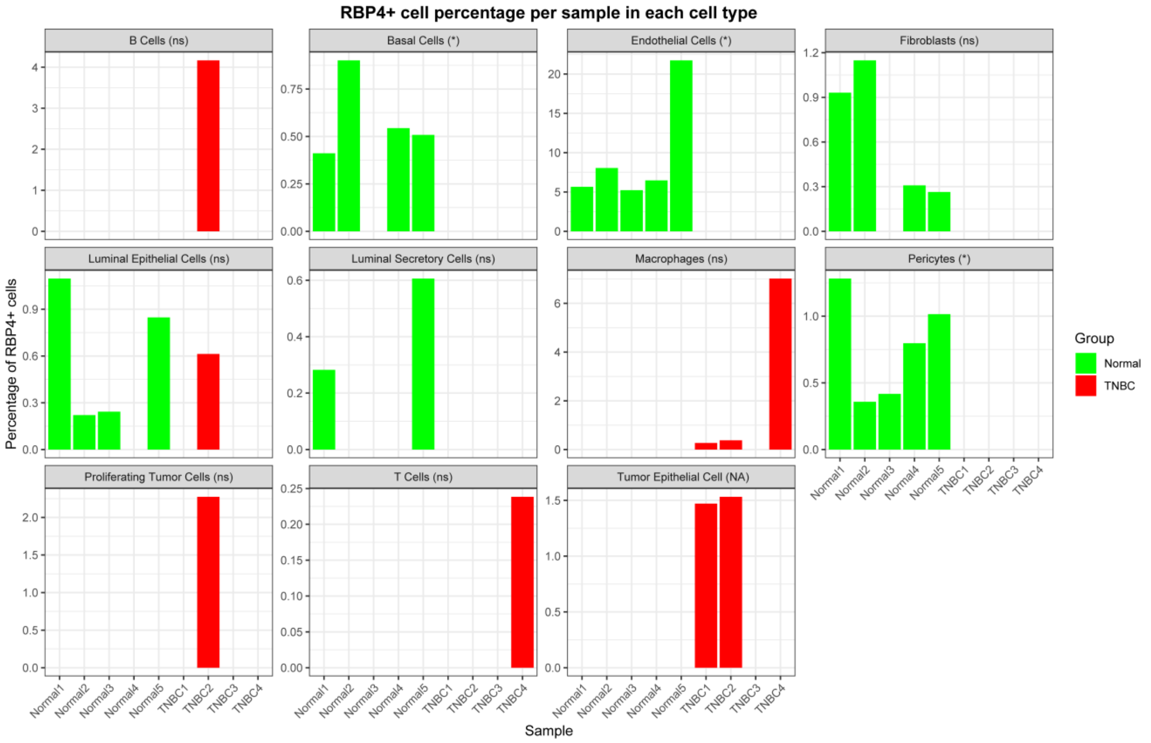

Figure S4. The percentage of RBP4 positive cells for each cell cluster. $*P < 0.05$; $**P < 0.01$; $***P < 0.001$.

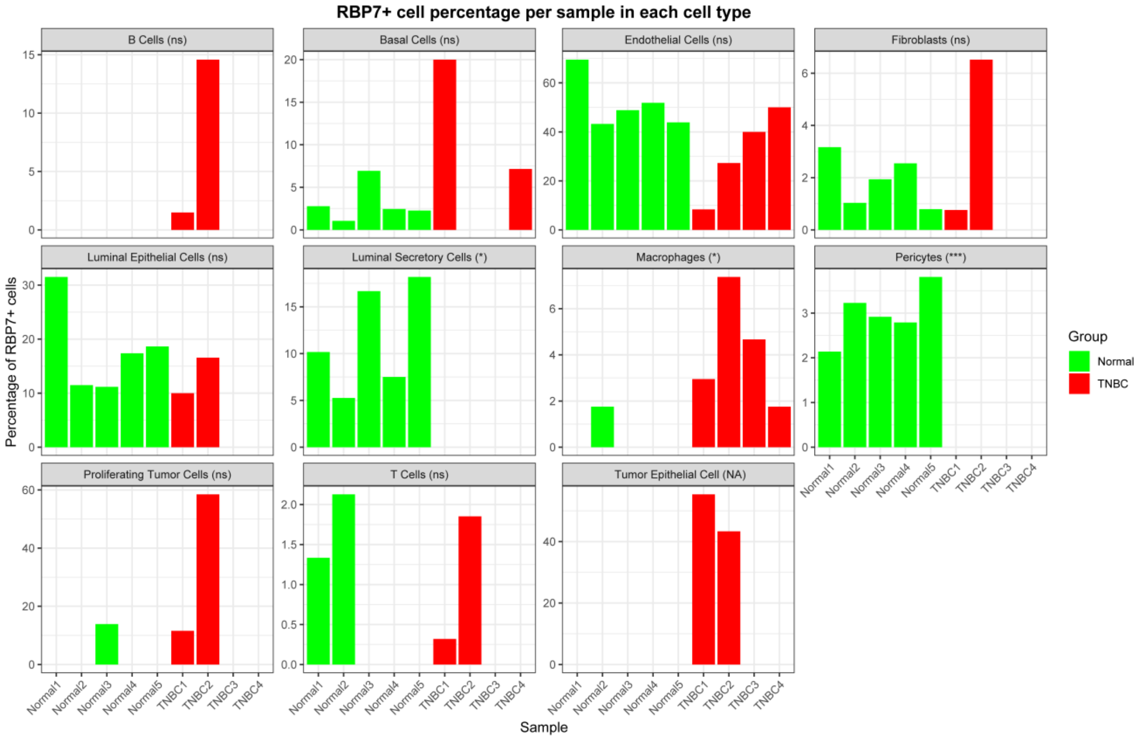

Figure S5. The percentage of RBP7 positive cells for each cell cluster. $*P < 0.05$; $**P < 0.01$; $***P < 0.001$.

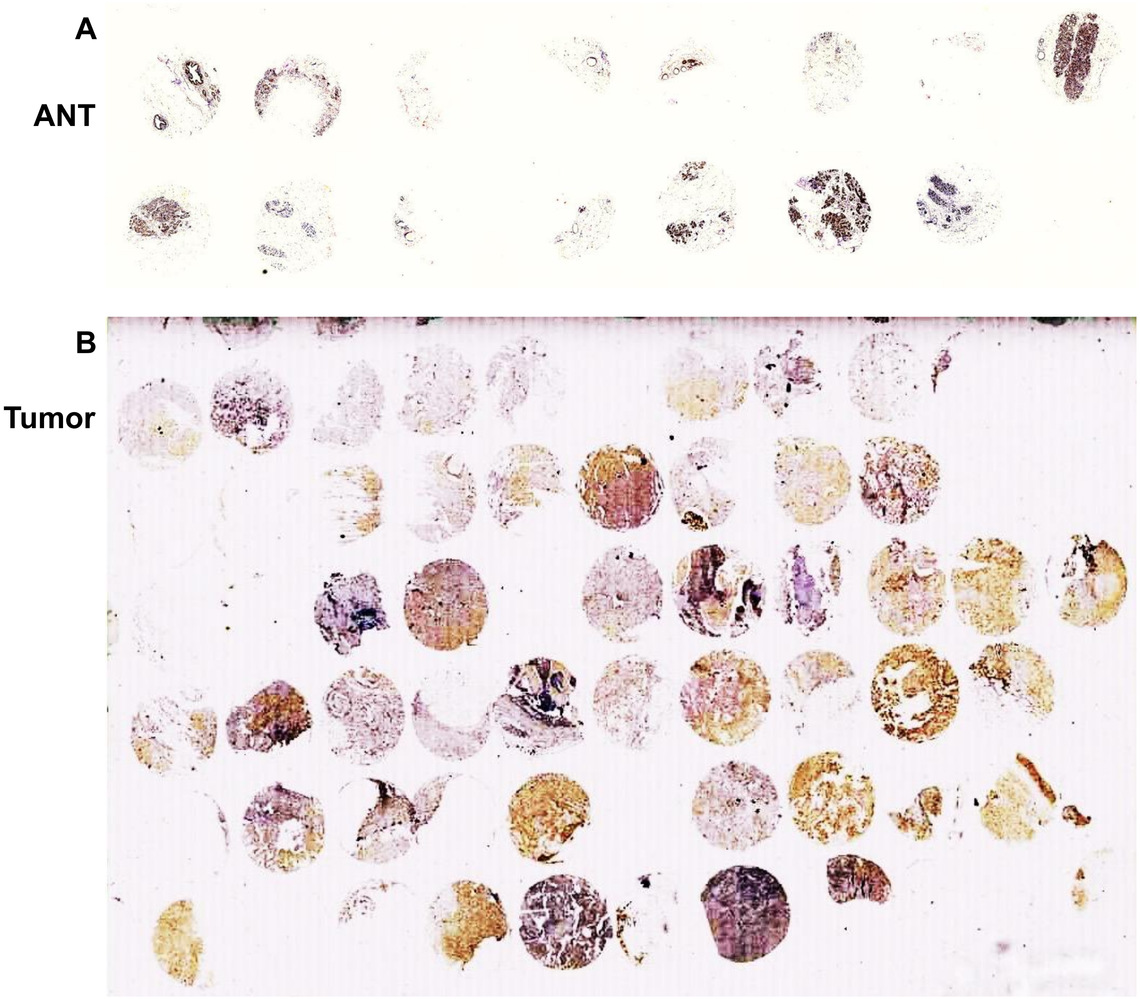

Figure S6. Tissue microarray staining (A) ANT group. (B) Tumor tissue group.
